# Wearable Sensors Based on Force-Sensitive Resistors for Touch-Based Collaborative Digital Gaming

**DOI:** 10.3390/s22010342

**Published:** 2022-01-04

**Authors:** Balaji Dontha, Kyoung Swearingen, Scott Swearingen, Susan E. Thrane, Asimina Kiourti

**Affiliations:** 1 Department of Electrical and Computer Engineering, The Ohio State University, Columbus, OH 43210, USA; kiourti.1@osu.edu; 2 Department of Design, The Ohio State University, Columbus, OH 43210, USA; swearingen.75@osu.edu (K.S.); swearingen.16@osu.edu (S.S.); 3 College of Nursing, The Ohio State University, Columbus, OH 43210, USA; thrane.2@osu.edu

**Keywords:** collaborative digital gaming, conductive threads, embroidery, force-sensitive resistor, wearable sensors

## Abstract

We report new classes of wearable sensors that monitor touch between fully-abled and disabled players in order to empower collaborative digital gaming between the two. Our approach relies on embroidered force-sensitive resistors (FSRs) embedded into armbands, which outperform the state-of-the-art in terms of sensitivity to low applied forces (0 to 5 N). Such low forces are of key significance to this application, given the diverse physical abilities of the players. With a focus on effective gameplay, we further explore the sensor’s touch-detection performance, study the effect of the armband fabric selection, and optimize the sensor’s placement upon the arm. Our results: (a) demonstrate a 4.4-times improvement in sensitivity to low forces compared to the most sensitive embroidered FSR reported to date, (b) confirm the sensor’s ability to empower touch-based collaborative digital gaming for individuals with diverse physical abilities, and (c) provide parametric studies for the future development of diverse sensing solutions and game applications.

## 1. Introduction

Over 5.5 million children in the United States have a cognitive or physical disability that results in at least some difficulty with activities, including play [1,2,3]. Example developmental disabilities include cerebral palsy (CP), autism spectrum disorder (ASD), muscular dystrophy (MD), and Down’s syndrome, etc. [4], and are often accompanied by physical impairments such as spasticity, muscle contracture, bone deformity, muscle weakness, and coordination disorders. These conditions cause deficits in day-to-day activities, such as grabbing or holding objects [5,6]. In turn, these children may lose their ability to play with their family, friends, or primary caregivers, and are less able to express themselves and make meaningful connections with others [1,7].

According to The American Academy of Pediatrics, play is essential to development because it contributes to the cognitive, physical, social and emotional wellbeing of children and youth [8]. Play has also been recognized by the United Nations High Commission for Human Rights as a right of every child [9]. Research in [10,11,12,13,14,15,16] indicates how cognitive abilities such as language, memory, self-regulation, and the ability to plan, focus and execute tasks can be improved with gaming. To date, several digital games have been developed for children with CP, ASD and other disabilities, but they are either single player or rely on a virtual assistant. For example, ‘A Sunny Day: Ann and Ron’s World’ uses an iPad game application [17], while ‘TeachTown: Basics’ adopts computer-assisted instructions and gamifies traditional treatment exercises into rewards to motivate learning [18]. Unfortunately, the concept of single-player games undermines the idea of collaborative gameplay. By contrast, projects like ‘Invasion of the Wrong Planet’ [19], ‘Collaborative Puzzle Game’ [20], and ‘SIDES’ [21] encourage collaboration (e.g., to defend a planet or solve a jigsaw puzzle). Nevertheless, they are only suitable for children with mild disabilities. For moderate developmental delays, commercial gaming platforms (X-box [22], Nintendo [23], etc.) are taking initiatives to adapt their consoles. However, these gaming consoles are usually challenging. Finally, for children with severe disabilities, communities like The AbleGamers [24], Special Effect [25] and Warfighter Engaged [26] take major steps, as does eye-gaze-based interaction integrated with Digital Games-Based Learning [27]. Nevertheless, games designed for children with severe disabilities tend to target learning instead of bonding with a parent or caregiver, which is an aspect that is critical to the child’s development [28].

In order to address shortcomings in the state-of-the-art, we propose collaborative games that rely on touch between fully-abled and disabled players, i.e., physical touch between the players is sent as an input for the gameplay. Wearable sensors capable of detecting touch play a key role in this regard. Here, we focus on FSRs as a reliable, cost-effective, and flexible solution [29]. We also focus on embroidered FSRs, as embroidered surfaces are known to be mechanically robust, tolerant to repetitive deformations, and washable. A key requirement for this FSR design is to be highly sensitive to low forces applied upon the sensor by individuals with disabilities; a performance metric that previously reported FSRs failed to meet. More specifically, Ref. [29] describes a significant decrease in gross and fine finger dexterity in children with ASD, Ref. [30] reports lower peak grasp forces for children with ASD compared to typically developing children, and Ref. [31] shows that children with ASD and MD have impaired lower-hand symmetry. With the touch force applied by healthy individuals ranging from 1.27 N to 3.22 N [32], the forces applied by children with disabilities are expected to be even lower. Referring to Table 1, most of the previously reported embroidered FSRs focus on wide dynamic ranges of employed forces (e.g., up to 20 N [33], 30 N [34], or 56.7 N [35]) and exhibit poor sensitivity in the detection of small forces. The work in [36] studies a narrow dynamic range of 0–5 N, but the resulting sensitivity is poor, which was attributed to the high resistivity of the employed threads. Relatively newer techniques other than FSR sensors highlighted in [37,38] use hydrogel elastomer ionic sensors for hand motion monitoring. However, the fabrication is sophisticated and expensive compared to FSR sensors. Likewise, there are potential problems with hydrogel dehydration and limited temperatures of operation. The previous solutions are, thus, unsuitable for the application under consideration.

In this paper, we report new classes of embroidered FSR sensors that are optimized for collaborative gameplay between individuals of various physical abilities, and which outperform the state-of-the-art in terms of sensitivity to low forces (<5 N). In order to optimize the experience of gameplay, we study the ergonomics of the proposed sensors by exploring various fabrics and various locations upon the human body. Section 2 presents the system architecture and provides details on the employed materials and methods. Section 3 reports our results, while Section 4 discusses our findings. The paper concludes in Section 5.

## 2. Materials and Methods

### 2.1. System Overview

**Figure 1:**
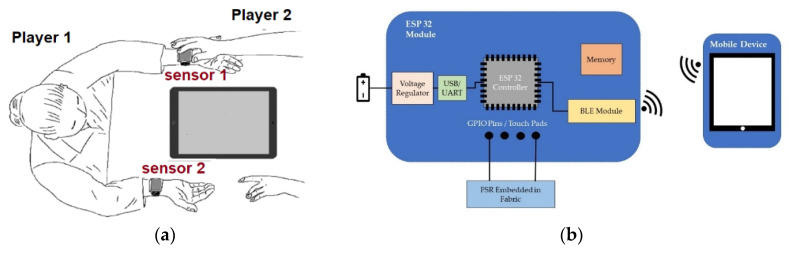
(**a**) Mechanism of gameplay using wearable sensors functionalized with FSRs; (**b**) block diagram of the proposed wearable sensor communicating wirelessly with a mobile device.

Figure 1a depicts the proposed mechanism of gameplay. The two players sit facing each other with a mobile device (e.g., a tablet) placed in-between them. Wearable sensors in the form of an armband are worn by either or both players, depending on their physical and cognitive abilities. The sensors are functionalized with FSRs that are specifically designed and optimized for this application (see Section 2.2), such that when a player touches the sensor, an input is sent wirelessly (e.g., via Bluetooth) to the tablet. This input may enable a frog to jump, a car to switch lanes, or a rocket to shoot, depending on the game’s design. Depending on the number of inputs the game is designed for, more than one armband may be employed. For example, in Figure 1a, Player 1 may be a child with disabilities and Player 2 may be a parent without disabilities, playing a game with a total of three inputs (touching sensor 1, touching sensor 2, and concurrently touching sensors 1 and 2). In this case, the passive mode of operation for Player 1 accommodates the entire spectrum of mild to severe disabilities.

**Figure 2:**
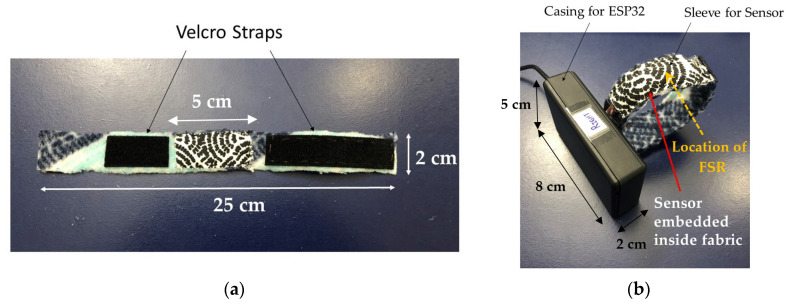
(**a**) Armband prototype with embedded FSR; (**b**) wearable sensor showing the casing for the ESP32 and the wristband with the FSR.

The block diagram of the proposed FSR-based sensor is shown in Figure 1b. The FSR is embedded in fabric, the selection of which is subject to optimization in this research. This fabric integration serves two purposes. First, it keeps the FSR in place in a comfortable manner for the wearer. Second, it prevents direct contact between the FSR and the human skin, preserving the FSR’s electrical properties and minimizing drifts in performance. An example implementation of an armband with embedded FSR is shown in Figure 2a. This FSR-functionalized armband is then connected to a microcontroller (in this case the ESP32 module developed by ESPRESSIF Systems) using the general-purpose input/output (GPIO)/touchpad pins; see Figure 2b. The ESP32 was chosen due to its low cost and power consumption. Whenever the FSR senses the touch, it wakes up the ESP32 from deep-sleep mode and stores the data in a register of the ESP32 memory. The process for the data storage and handling is explained in [39,40]. Data from the ESP32 memory are then sent to a mobile device via Bluetooth Low Energy (BLE) [41], and are used to control the mechanics of the game. For power, we connect the microcontroller to a power bank via a USB cable. However, batteries can be used instead. Specifically, the ESP32 microcontroller operates at 3.3 V and has 500 mA of current handling capacity [42]. The BLE component consumes 130 mA to transmit data and 95–100 mA to receive data. This corresponds to 0.429 W of power consumed. Considering 1 h of active gameplay, we consume 130 mAh of power. When the ESP32 is used in deep-sleep mode, the current consumption is 150 μA, resulting in 0.495 mW or 0.15 mAh of power consumption, which significantly saves battery life and increases play time.

### 2.2. Research Design

The step-by-step methodology pursued in this work is outlined below.

**Figure 3:**
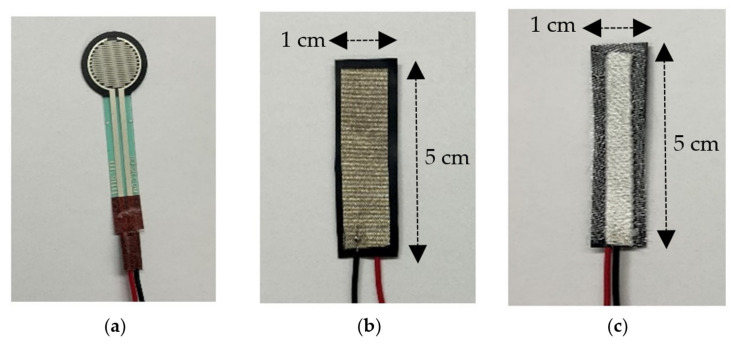
FSR designs explored in this work: (**a**) off-the-shelf FSR; (**b**) thru-mode FSR with woven conductive fabric; (**c**) thru-mode FSR with embroidered e-threads.

**Figure 4:**
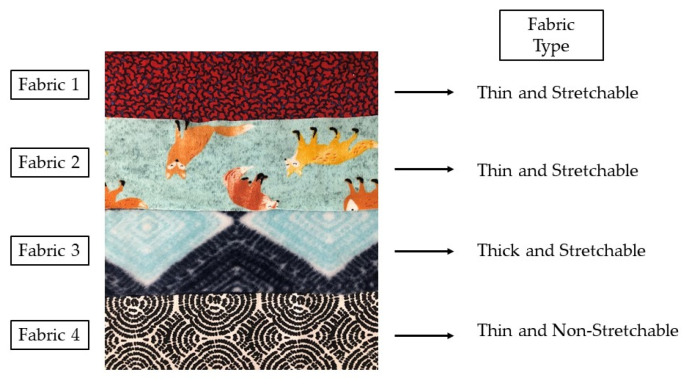
Fabrics tested for the embedding of the FSR and the realization of the wristband for touch-based collaborative digital gaming.

First, in Section 3.1, we confirm the need to integrate the FSR in fabric (rather than exposing it directly to the skin) and optimize the fabric selection. In order to lower risk in this first stage of validation, and without a loss of generality, we use an off-the-shelf shunt-mode FSR which is circular in shape, with a diameter of 12.5 mm (see Figure 3a) [43]. In the selection of the fabric, both the thickness and elasticity are of relevance to this application. The thickness relates to the isolation between the FSR and the skin, while elasticity relates to the potential deformations/wrinkles on the sensors that could cause false positives. With these in mind, we test four types of fabrics (see Figure 4) that vary in terms of thickness and elasticity: Fabric 1 is a polyester, stretchable, 0.35-mm-thick fabric; Fabric 2 is a cotton, stretchable, 0.54-mm-thick fabric; Fabric 3 is a polyester, stretchable, 0.82-mm-thick fabric; and Fabric 4 is a cotton, non-stretchable, 0.38-mm-thick fabric. The ESP32 register values are recorded during ‘no touch’ and ‘touch’ to identify the setup resulting in the maximum changes of the registered values.

Second, in Section 3.2, we explore the optimal placement of the FSR-functionalized armband, considering the gameplay scenario of Figure 1a. This is critical to evaluate, as our sensor is intended for specially-abled children with limited limb movement and a compromised ability to apply force. Similarly to the above, and without a loss of generality, we use the same off-the-shelf shunt-mode FSR of Figure 3a and place it upon three different locations, i.e., the forearm, the palmar side of the hand, and the dorsal side of the hand. The ESP32 register values are again recorded during ‘no touch’ and ‘touch’ to identify the location resulting in maximum changes of the register values.

**Figure 5:**
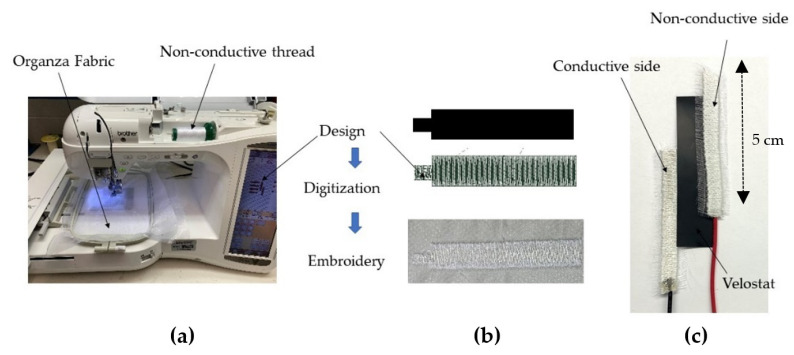
Embroidery Process with a Brother 4500D Embroidery Machine, outlining the formation of a thru-mode embroidered FSR. (**a**) embroidery machine; (**b**) embroidery process; (**c**) thru-mode embroidered FSR.

Third, in Section 3.3, we demonstrate the superiority of textile-based FSRs optimized for this particular application and fabricated in-house, and particularly an embroidered FSR (see Section 3.3). Specifically, a major limitation in the case of the off-the-shelf prototype of Figure 3a is that it is not mechanically robust. That is, the FSR can easily break or permanently deform over the course of time/gameplay, losing its functionality. This prototype is also not washable/dryable, which is a major inconvenience for a textile-based sensor. Finally, as demonstrated in our Results section, this off-the-shelf sensor is by no means optimized for the application under consideration, leading to an increased number of false positives. In order to address the shortcomings above, we expand our study with two in-house FSRs. We pursue Thru-mode FSRs with a Velostat placed in-between two conductive plates, with the potential to be implemented fully on textiles. Both FSRs are 5 cm × 1 cm in size, selected so as to maximize the sensing area when integrated into an armband:

- The first implementation shown in Figure 3b uses woven conductive fabric from Adafruit Industries, LLC made of copper, and nickel-plated polyester with a surface resistivity of 0.05 Ω/sq [44]. Velostat of 0.1 mm thickness is sandwiched between the two conducting sheets using a permanent fabric adhesive to form the FSR.
- The second implementation shown in Figure 3c relies on the embroidery of seven-filament silver-plated copper Elektrisola e-threads exhibiting a very low resistivity of 1.9 Ω/m and a fine diameter of 0.12 mm. These e-threads are placed in the bobbin case of an automated Brother 4500D embroidery machine, whereas the non-conductive polyester threads are placed in the spool pin. Referring to Figure 5, the target design is first digitized (i.e., the path of the needle is determined) and then embroidered in an automated manner. As discussed in Section 3, the selection of high-conductivity e-threads (see the resistivity comparison in Table 1) is the key to boosting the FSR’s sensitivity at low applied forces. Similarly to the above, we prototype two conductive sides and adhere Velostat in-between them using fabric adhesive. We expect that the improved conductivity of the embroidered (vs. the woven) surface, as has been extensively validated in the past [45,46], will improve the FSR’s performance. E-threads are also known to be much more tolerant to mechanical deformations, high/low temperatures, and laundering, adding to the superiority of the embroidered FSR.

Finally, in Section 3.4, we explore the trade-offs associated with the varying embroidered densities of the FSR shown in Figure 3c. We identify an optimal embroidery density value while also providing an extensive discussion and experimental results to guide future implementations.

## 3. Results

### 3.1. Optimization of the Fabric Selection

As a proof-of-concept, we used the off-the-shelf FSR of Figure 3a and placed it on the forearm, in direct contact with the skin, and when embedded within each of the four fabrics of Figure 4. Table 2 shows the ESP32 register values for the ‘no touch’ and ‘touch’ cases, including touches with one finger, two fingers, the palmar side of the hand, and the dorsal side of the hand. In order to quantify these touches, we estimate the one-finger touch to an average applied force of 1 N. Similarly, the two finger, palmer side and dorsal side of the hand are estimated to an average applied force of 2 N, 4 N and 6 N, respectively.

Referring to Table 2, when someone touches the FSR, the voltage drops across the touch pin of the ESP32 microcontroller, causing the values in the ESP32 register to drop as well. It can be observed in Table 2 that the low thicknesses of Fabrics 1 and 4 provide the poor isolation of the FSR sensor from the skin, causing poor dynamic range. Fabrics 2 and 3 provide better dynamic range. Thick fabrics are thus preferred. Taking all of the listed touches into account, the maximum change in register values for ‘touch’ vs. ‘no touch’ is the highest when using the stretchable thick fabric, referred to as Fabric 3 in Figure 4. This is because its thickness provides good isolation from the skin, while its elasticity preserves the shape of the FSR. Thick and elastic fabrics were, hence, considered for the further analysis with textile-based FSRs for the proposed application, and we proceeded with the embedding of the textile FSR sensor in Fabric 3.

### 3.2. Optimization of the Sensor Placement

When the sensor is placed on curved surfaces such as an arm, the sensor deforms, resulting in decreased sensitivity to applied forces. It is thus crucial to study the performance of the FSR sensor on different curvatures, as highlighted in Table 3. In order to account for different curvatures and understand the effect on the FSR performance, we again considered the off-the-shelf FSR of Figure 3a and placed it upon three different locations, i.e., the forearm, palmar side of the hand, and dorsal side of the hand. The ESP32 register values were recorded during ‘no touch’ and ‘touch’, and are summarized in Table 3. Here, we selected one-finger touch as a worst-case scenario (i.e., gentle touch ~1 N of force) to help assess the sensor’s sensitivity. As seen, Fabric 3 provides the best dynamic range, further validating our conclusion from Table 2. The placement of the sensor on the forearm provides poor results compared to the palmer/dorsal side of hand, especially when placed directly on the skin. This is because the sensor is less conformal, implying that a relatively flat/uniform surface is better to place the sensor on. It was observed that the placement of the FSR sensor on the palm-side of the hand provides the best performance, followed by placement on the forearm, and then placement on the dorsal side of the hand. This is because sensor deformation degrades the performance, resulting in the poor sensing capabilities of the FSR, as is also discussed in Table 2. In our case, we target gameplay for children with disabilities who may have contracted limbs (e.g., arms, hands and fingers curled inwards), and, thus, proceed with placing the FSR sensor on the forearm. Of course, this is not limiting, and may vary per application scenario.

### 3.3. Demonstration of the Improved Sensitivity of the Embroidered FSR

**Figure 6:**
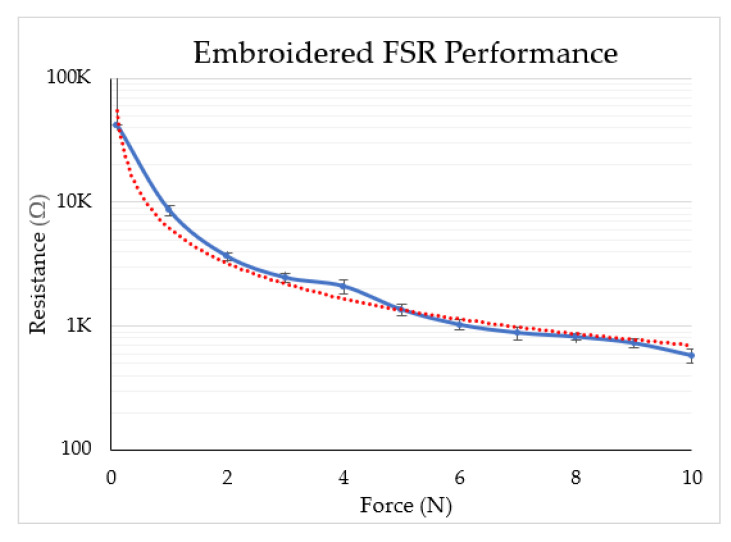
Sensitivity of the embroidered FSR up to 10 N.

The performance of the embroidered FSR was measured under an applied force of up to 10 N, and the results are plotted in Figure 6. Because the objective is to measure small forces, we calculated the sensitivity of the FSR up to 5 N using the equation given in (1). The applied force was changed from 0 N to 5 N, and the corresponding change in the resistance value of the FSR was measured. The embroidered FSR is non-linear up to 2 N, and then exhibits a relatively linear relationship up to 10 N. The maximum deviation of the resistance value from the nominal resistance for an applied force is 12% at 4 N, while for all other cases, the deviation is less than 10%. The reliable dynamic range of the embroidered FSR sensor is up to 20 N. However, we plotted the data only up to 10 N, as the range of operation for touch-based operation is less than 10 N. The dynamic range and sensitivity can be altered by adjusting the stitching density of the embroidery. Compared to previously reported embroidered FSRs (see Table 1), our embroidered FSR exhibits significantly higher sensitivity. As such, it can be readily implemented to detect small forces, such as one-finger touch.

(1) $$\mathrm{Sensitivity}\;\mathrm{of}\;\mathrm{FSR}=\frac{\mathrm{Rmax}-\mathrm{Rmin}}{\mathrm{Change}\;\mathrm{in}\;\mathrm{applied}\;\mathrm{force}}=\frac{41.992k-1.355k}{5-0}\;=8073.4\;k\Omega /N$$

We embedded the three FSRs in Fabric 3, placed the resulting armband on the forearm, and recorded the ESP32 register values during ‘no touch’ and ‘touch’ in Table 3. Similarly to Table 1, four different types of touch were considered in order to account for different mechanics of gameplay. Referring to Table 4, textile-based FSRs are promising substitutes to off-the-shelf FSR sensors. Both woven and embroidered FSRs exhibit a dynamic range that is suitable for differentiation between ‘no touch’ and ‘touch’ cases. Nevertheless, the embroidered FSR considerably outperforms the woven FSR and the off-the-shelf FSR via an impressive dynamic range that can minimize false positives. In order to further explore the latter, we pursued 50 trials where we randomly touched the sensor in different orientations and configurations outlined in Table 5. This is of critical importance for the target population, as players with motor disabilities may not be able to precisely touch the sensor. As expected, the error rates associated with the embroidered FSR are much smaller than those of the woven FSR and the off-the-shelf FSR. The reason for the significant improvement in the embroidered FSR is the very low resistivity of the e-threads, as indicated in Section 2.2. The ability to control the stitch density based on the application allows the improvement in flexibility and conformability of the FSR sensor, reducing the number of false positives, as highlighted in Table 4.

### 3.4. Optimization of the Embroidery Density

Finally, we remark that the embroidered FSR of Table 4 and Table 5 is realized using a density of 4 e-threads/mm. This density selection is justified in Table 6, in which three embroidered FSRs of different e-thread densities are compared: 1 e-thread/mm, 4 e-threads/mm, and 7 e-threads/mm. The experimental setup embedded these three embroidered FSRs in Fabric 3, placed the bands on the arm, and evaluated ‘no touch’ vs. ‘one-finger touch’ cases. As seen, the embroidery density of 4 e-threads/mm performs the best, as it is an optimal compromise between conductivity and mechanical performance. Specifically, at 1 thread/mm, the surface conductivity of the FSR is poor. At 7 e-threads/mm, the surface conductivity is improved, but the resulting thickness and stiffness of the FSR increase the chance of the top and bottom conductor pressing against each other. In turn, this lowers the cut-off for the ‘no touch’ scenario, degrading the sensitivity to touch.

## 4. Discussion

The proposed wearable sensors functionalized with FSRs provide a promising solution to enable collaborative digital gaming and other touch-based solutions. The results indicate that textile-based FSRs can replace off-the-shelf FSRs in this regard to make the sensor more seamless and durable. In particular, embroidered FSRs provide a high level of control over the resulting conductivity and mechanical performance, enabling optimized sensors with minimal false positives in the detection of ‘touch’ vs. ‘no touch’. Regardless of the FSR selection, a need was demonstrated to separate the sensing element from the human skin. Fabrics were explored to this end that considered thickness and stretchability factors to optimize performance. It was found that thick and stretchable fabrics work the best. It can be noted that the studies reported in Table 2 and Table 3 were performed using off-the-shelf FSRs, which account for the worst-case scenario. In-house FSRs (woven fabric/embroidered) can be designed to be conformal to the arm, i.e., the forearm, the palmar/dorsal side and so on, as per the requirements of the user, thus reducing the errors seen in off the shelf FSRs. Fabric 4 was chosen in the same way because of the skin isolation it provided. As a result, the performance of any type of FSR on a similar fabric type would be identical.

The resulting armband can then be designed in a form factor that fits the application needs under consideration. For example, we selected a small width of 2 cm for the armband of Figure 2a to minimize the fabric coverage upon the arm and enhance skin-to-skin contact between the players. Similarly, the sensor placement upon the human body may vary per the application needs, though the performance was shown to improve upon flat/uniform areas. Various types were also explored, and the robustness of the idea was confirmed in all cases. Overall, multiple possibilities can be explored should the designer have a specific game application and target demographic in hand.

On a system level, ‘touch’ and ‘no touch’ inputs can be registered on an ESP32 microcontroller and transmitted wirelessly via Bluetooth to a remote mobile device (e.g., a tablet). ESP32 in the deep-sleep mode and BLE mode of operation are ideal for reducing power consumption and increasing the time of play. Though we have experimented with a power bank, Li-Ion or Li-Po batteries are also suitable. These batteries are rechargeable, and range from 150 mAh to 2500 mAh.

In the future, we can explore more seamless sensor designs by implementing shunt-based FSRs on embroidered e-threads, and by designing in-house electronics. Textile-based piezo-resistive materials can also be explored to replace the Velostat, and to enable fully-textile substitutes for the FSR sensors. The mechanical/thermal performance and launderability will also be explored for the sensors. Finally, we have the option to use multiple FSR sensors to further expand the sensing area and potentially improve the resulting sensitivity.

## 5. Conclusions

New classes of wearable sensors functionalized with FSRs were reported for touch-based collaborative gaming. An off-the-shelf FSR was originally selected and connected to an ESP32 microcontroller to ultimately transfer data to a remote tablet in a wireless manner. The placement of the sensor directly on the skin was found to compromise performance, and necessitated fabrics to be placed in between. Four different types of fabrics were tested in this regard, indicating that thick and stretchable options were the most suitable. Multiple on-body locations were analyzed for the sensor, and placement on the palm side of the hand was identified as optimal, followed by placement on the forearm. Depending on the application and target audience, different placements can be considered. Different touch scenarios were also explored to consider players with physical disabilities. Finally, textile-based FSRs were explored, including an embroidered version that was shown to considerably outperform the rest in terms of sensitivity to low applied forces. The stitching density was altered during the embroidery process to identify an optimal value for effective gameplay.

As an example application, this work intends to enhance the bonding between children with disabilities and their parents without disabilities. In the future, we plan to use the FSR sensors in a real-world game environment, connected to an iPad or other Bluetooth-enabled device. The study aims to evaluate the performance of the fabricated FSR sensors compared to commercial sensing systems. However, numerous other applications may be considered for diverse age groups and/or medical conditions. Our sensors can also be expanded to textile-based force-sensing alternatives, such as pressure sensing mats for bed-bound patients, or pressure sensitive socks for sprinters and marathon runners.

## Figures and Tables

**Table 1:** Comparison of the reported solution compared to previously reported embroidered FSR.

Ref.	Application	Dynamic Range Explored	Sensitivity ^1^ (Change in Resistance) from 0 to 5 N	E-Thread Resistivity (Ω/m)
This work	Collaborative gaming	<5 N	>8000	1.9
[33]	Driving gloves	0–20 N	80	127
[34]	Object Recognition	0–30 N	1000	200 (Ω/m^2^)
[35]	Respiration/Posture Monitoring	0.56–56.7 N	40	-
[36]	Mobility Detection	0–5 N	1800	120

^1^ The calculation of the sensitivity is explained in Section 3.3.

**Table 2:** Performance of off-the-shelf FSR for different types of touches when placed on the forearm.

		Value in ESP32 GPIO/Touch Pin Register				
		Direct Skin	Fabric 1	Fabric 2	Fabric 3	Fabric 4
no touch	(a)	15–18	23–24	26–27	26–27	24–26
1-finger touch	(b)	0–2	1–3	1–2	1–2	1–2
2-finger touch	(c)	0–1	1–2	1–2	0–1	1–2
palmar side of hand touch	(d)	0–1	1–2	1–3	0–2	1–2
dorsal side of hand touch	(e)	0–2	1–3	1–3	1–2	1–3
Max. change in register value	(c)–(a)	14–18	21–23	24–26	25–27	22–25

**Table 3:** Performance of the off-the-shelf FSR for one-finger touch when placed on different locations.

			Value in ESP32 GPIO/Touch Pin Register				
			Direct Skin	Fabric 1	Fabric 2	Fabric 3	Fabric 4
forearm	no touch	(a)	15–18	23–24	26–27	26–27	24–26
	1-finger touch	(b)	0–2	1–3	1–2	1–2	1–2
palmar side of hand	no touch	(c)	13–16	24–25	26–27	28–29	27–28
	1-finger touch	(d)	0–1	1–2	0–2	1–2	1–2
dorsal side of hand	no touch	(e)	24–25	24–26	25–27	26–27	26–28
	1-finger touch	(f)	0–2	0–2	0–1	1–3	1–2
Max. change in register value			22–25 (e)–(f)	22–26 (e)–(f)	24–27 (c)–(d)	26–28 (c)–(d)	25–27 (c)–(d)

**Table 4:** Performance comparison of the three FSRs shown in Figure 3.

		Value in ESP32 GPIO/Touch Pin Register		
		Off-the-Shelf FSR	Woven Fabric FSR	Embroidered FSR
no touch	(a)	26–27	19–21	57–58
1-finger touch	(b)	1–2	0–2	0–1
2-finger touch	(c)	0–1	0–1	0–1
palmar side of hand touch	(d)	0–2	0–1	0–1
dorsal side of hand touch	(e)	1–2	0–1	0–1
Max. change in register value	(a)–(c)	25–27	18–21	56–58

**Table 5:** Error calculation for different touch patterns.

		Off-the-Shelf FSR		Woven Fabric FSR		Embroidered FSR	
	Trials	No. of Touches Registered	% Error	No. of Touches Registered	% Error	No. of Touches Registered	% Error
1-finger precision touch	50	48	4%	47	6%	49	2%
2-finger precision touch	50	49	2%	46	8%	49	2%
1-finger partial touch	50	45	10%	46	8%	47	6%
2-finger partial touch	50	43	14%	44	12%	48	4%
random palm touch	50	49	2%	47	6%	49	2%
center of palm touch	50	26	48%	36	28%	42	16%

**Table 6:** Effect of the e-thread density on the performance of embroidered FSRs.

	Value in ESP32 GPIO/Touch Pin Register		
	1 e-Thread/mm	4 e-Threads/mm	7 e-Threads/mm
no touch	43–44	49–50	39–41
1-finger touch	19–21	0–1	0–1
Max. change in register value	22–25	48–50	38–41
